# Efficient targeted mutagenesis in tetraploid *Pogostemon cablin* by the CRISPR/Cas9-mediated genomic editing system

**DOI:** 10.1093/hr/uhae021

**Published:** 2024-01-16

**Authors:** Shiqiang Xu, Fangping Li, Fang Zhou, Jingyu Li, Shike Cai, Shaohai Yang, Jihua Wang

**Affiliations:** Guangdong Provincial Key Laboratory of Crops Genetics and Improvement, Crops Research Institute, Guangdong Academy of Agricultural Sciences, Guangzhou, 510640, China; Guangdong Provincial Engineering and Technology Research Center for Conservation and Utilization of the Genuine Southern Medicinal Resources, Guangzhou, 510640, China; Guangdong Provincial Key Laboratory of Crops Genetics and Improvement, Crops Research Institute, Guangdong Academy of Agricultural Sciences, Guangzhou, 510640, China; Guangdong Provincial Engineering and Technology Research Center for Conservation and Utilization of the Genuine Southern Medicinal Resources, Guangzhou, 510640, China; Guangdong Provincial Key Laboratory of Crops Genetics and Improvement, Crops Research Institute, Guangdong Academy of Agricultural Sciences, Guangzhou, 510640, China; Guangdong Provincial Engineering and Technology Research Center for Conservation and Utilization of the Genuine Southern Medicinal Resources, Guangzhou, 510640, China; Guangdong Provincial Key Laboratory of Crops Genetics and Improvement, Crops Research Institute, Guangdong Academy of Agricultural Sciences, Guangzhou, 510640, China; Guangdong Provincial Engineering and Technology Research Center for Conservation and Utilization of the Genuine Southern Medicinal Resources, Guangzhou, 510640, China; Guangdong Provincial Key Laboratory of Crops Genetics and Improvement, Crops Research Institute, Guangdong Academy of Agricultural Sciences, Guangzhou, 510640, China; Guangdong Provincial Engineering and Technology Research Center for Conservation and Utilization of the Genuine Southern Medicinal Resources, Guangzhou, 510640, China; Guangdong Provincial Key Laboratory of Crops Genetics and Improvement, Crops Research Institute, Guangdong Academy of Agricultural Sciences, Guangzhou, 510640, China; Guangdong Provincial Engineering and Technology Research Center for Conservation and Utilization of the Genuine Southern Medicinal Resources, Guangzhou, 510640, China; Guangdong Provincial Key Laboratory of Crops Genetics and Improvement, Crops Research Institute, Guangdong Academy of Agricultural Sciences, Guangzhou, 510640, China; Guangdong Provincial Engineering and Technology Research Center for Conservation and Utilization of the Genuine Southern Medicinal Resources, Guangzhou, 510640, China

Dear Editor,

Patchouli [*Pogostemon cablin* (Blanco) Benth.], a species in the *Pogostemon* genus of the Lamiaceae family, is an aromatic herb with significant medicinal and industrial value. The dried aerial parts of patchouli, known as *guang huo xiang* in traditional Chinese medicine, were a genuine medicinal material in southern China and officially recorded in the Chinese Pharmacopoeia. As an aromatic dehumidifying medicine, patchouli was clinically used to treat colds, nausea, diarrhea, headache, and fever [1]. Patchouli has attracted worldwide attention as an antiviral drug to prevent the current global epidemic of coronavirus disease 2019 (COVID-19) [2, 3]. The flowering and seeding of patchouli are sporadic, and it is propagated by cuttings in cultivation. Asexual reproduction reduces the genetic diversity of patchouli, making it impossible to develop new germplasm and varieties through traditional crossbreeding. In addition, long-term asexual reproduction causes germplasm degradation, resulting in unstable quality of medicinal materials and decreased resistance to diseases and pests [4]. Therefore, improving patchouli’s quality and disease resistance through molecular genetic improvement has become a top priority.

**Figure 1:**
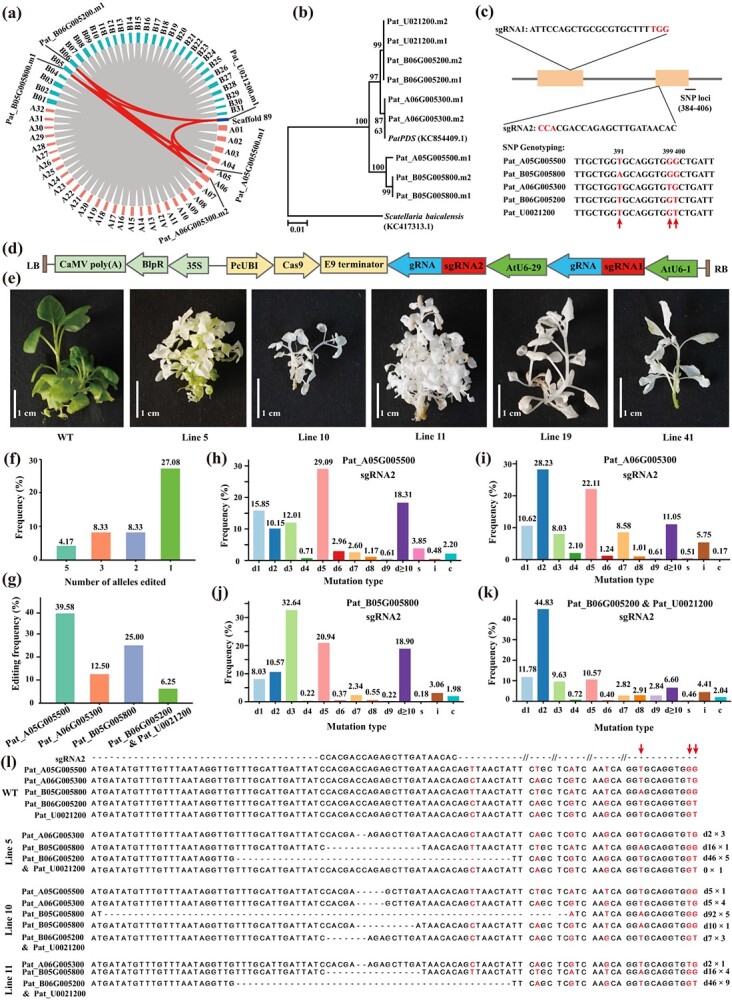
Targeted mutagenesis of PatPDS alleles by using PcUbi-pEGCas9 in tetraploid patchouli. (a) Duplication events of *PatPDS* genes in patchouli. Gray lines represent all the synteny gene pairs and red lines represent PatPDS gene pairs. (b) A maximum likelihood phylogenetic tree was constructed using the PDS protein sequences from patchouli and *Scutellaria baicalensis* in MEGA-X. m1 and m2 indicate alternative splicing of the *PatPDS* genes. (c) Schematic illustrating the PatPDS alleles with the two target sites located in the first and second exons. SNP sites near sgRNA 2 (sites 391, 399, and 400) were used for PatPDS genotyping. (d) Schematic diagram of PcUbi-pEGCas9 expression vector construction. (e) Phenotypes of PatPDS gene-editing mutants and wild-type plants. Lines 5 and 41 were chimeric phenotype, and Lines 10, 11, and 19 were albino phenotype. (f) Statistics of transgenic patchouli plants with edited *PatPDS* alleles. The number of alleles represents the event of editing *PatPDS* alleles in the same transgenic plant. The editing frequency was calculated by the number of *PatPDS* allele editing events by the total number of transgenic plants. (g) Editing efficiency of thoroughly editing different *PatPDS* alleles based on SNP genotyping. (h-k) CRISPR/Cas9-induced mutation types and frequency in *PatPDS* alleles. ‘d’ represents deletion, ‘i’ represents insertion, ‘s’ represents substitution, and ‘c’ represents the combination mutation. (l) Large fragment deletions in Lines 5, 10, and 11. ‘d’ represents deletion, and numbers indicate mutated bases for the corresponding target site and individual clones.

In patchouli, leaf explants have been transformed with *Agrobacterium tumefaciens* EHA101 harboring β-glucuronidase (*GUS*) and hygromycin phosphotransferase (*HPT*) genes, but the transformation process was largely inefficient due to the inhibitory effect of patchouli oil accumulated in leaves on *Agrobacterium* infection [5, 6]. A tetraploid hybrid and an immature genetic transformation system have hindered molecular biology research and genetic improvement in patchouli. CRISPR/Cas9-mediated genome editing technology holds great promise for genetic improvement, verification of gene function, regulation of plant metabolic networks, and generation of new germplasm. However, there have been no reports on the heritable applications of genome editing for patchouli. The availability of the genome of patchouli (a tetraploid hybrid consisting of two distinct subgenomes) affords the opportunity to utilize the CRISPR/Cas9 system [7]. Here, an efficient CRISPR/Cas9-based genome editing system was successfully established, and its effectiveness was verified by targeted knockout of the patchouli phytoene desaturase (*PatPDS*) allelic genes to produce albino plants.

Developing genetic manipulation tools requires an efficient transformation process and a precise genome editing system, while a plant regeneration system is a prerequisite for genetic transformation. Our preliminary experiments have shown that using leaves as explants can induce callus proliferation. However, the explants rapidly turned brown and became black, and the callus appeared white and watery, rendering it unsuitable for *Agrobacterium* infection. We selected tender buds with low essential oil content as explants, compared the efficacy of various hormone combinations through preliminary experiments, and established a suitable method for patchouli callus induction and differentiation. Under the optimized regeneration medium, the callus induction and differentiation rates reached 85 and 100%, respectively, indicating that the optimized hormone combination was suitable for patchouli genetic transformation.

*PDS* is a commonly used visual marker gene for testing gene editing tools in plants, and disruption of its function leads to albino phenotype [8]. To identify the *PDS* homologous alleles in tetraploid patchouli, a BLAST alignment was performed with *PatPDS* (KC854409.1) as a reference sequence. We found that five copies of the *PDS* gene with nine alternative splices were matched in the patchouli genome, indicating that the *PDS* gene undergoes duplication events in patchouli (Fig. 1a and b). These genes were distributed on four chromosomes and scaffold 89, respectively, and the coding regions were highly conserved, including 14 exons and 13 introns. Among them, the Pat_B06G005200 and Pat_U0021200 genes were highly conserved, with only one base difference at the 199 position upstream of the start codon. Then, these *PatPDS* genes were cloned from the cultivar ‘Zhao Xiang’ using specific primers PatPDS-F and PatPDS-R. To ensure the successful editing of the *PatPDS* allele, two target sites (sgRNA1 and sgRNA2) were designed near the 5′ end conserved region of these *PatPDS* genes, which were located in exon 1 and exon 2, respectively (Fig. 1c). Considering the challenge of homologous alleles for genotyping, we selected SNP sites near the sgRNA2 (391, 399, and 400 sites) for subsequent SNP genotyping (Fig. 1c). The CRISPR/Cas9-based genome editing system relies on a single guide RNA (sgRNA) to guide *Cas9* nuclease to the target site for gene editing. Therefore, the system’s efficiency is affected by the promoter that drives sgRNA expression [9]. To enhance the expression of sgRNA and improve the efficiency of gene editing, the transcription of the sgRNA expression cassette containing two target sequences driven by *Arabidopsis* promoters AtU6-1 and AtU6-29 and the parsley ubiquitin promoter (*PcUbi*) with higher activity than the CaMV 35S promoter were employed to drive the expression of *Cas9* endonuclease gene, respectively [10]. The constructed CRISPR/Cas9 binary vector with the Basta herbicide resistance gene *bar* as the marker gene was named PcUbi-pEGCas9 (Fig. 1d).

An appropriate concentration of Basta is vital for screening resistant callus to obtain transgenic plants. The selection pressure was optimized by the growth state of wild-type callus on induction medium supplemented with different concentrations of Basta. After 4 weeks of continuous culture, most calluses browned and died under 5 mg/l Basta, indicating that 5 mg/l Basta was the best pressure for screening transgenic calluses. The CRISPR/Cas9 vector was transformed into the patchouli friable embryogenic calluses using the optimized *Agrobacterium*-mediated transformation protocol. The plants regenerated from callus blocks showed green, albino, and chimeric phenotypes, of which the white and chimeric phenotypes accounted for 38.67 and 15.47%, respectively. Compared with typical plants, the chimeric plantlets were inlaid with white and green (line 5 and line 41), while some albino seedlings were dwarfed (Fig. 1e). To identify transgenic plants, 55 independent plants with albino (43 plants) and chimeric (12 plants) phenotypes were randomly selected to extract genomic DNA, and the putative gene-edited plants were identified by amplifying the *Cas9* gene. In general, the *Cas9* gene was not detected in four albino plants and three chimeric plants. These albino plants without the amplified *Cas9* gene may be attributed to the chloroplast genome aberration caused by somatic clonal variation in *vitro* culture. The remaining 48 plants were confirmed to be transgenic plants (87.27%), indicating that the *Agrobacterium*-mediated genetic transformation protocol was efficient and stable.

To verify the efficiency of the CRISPR/Cas9 system for targeted editing of the *PatPD*S alleles in patchouli, 48 transgenic plants, and one wild-type plant were high-throughput sequenced using primers flanking the target sites, and genotyping was based on the SNP polymorphism. All transgenic plants contained mutation at the sgRNA2 target site, while no plants showed any mutation at the site sgRNA1. The AtU6-1 promoter may not be able to drive the expression of sgRNA in patchouli or the inefficiency of the sgRNA1 site. To assess the editing efficiency between homologous alleles in polyploid chromosomes, we developed PolyDetecter to quantify the editing types between alleles by detecting allele-specific SNP sites. Among these 48 transgenic plants, two (4.17%) showed no wild-type allele, suggesting that all *PatPDS* alleles were mutated entirely. In addition, three alleles were eliminated in four plants (8.33%), two alleles were completely knocked out in four plants (8.33%), and 13 plants (27.08%) had only one allele eliminated (Fig. 1f). Based on SNP genotyping, 19 transgenic plants exhibited no wild-type sequence of the Pat_A05G005500 gene, indicating that the editing efficiency was 39.58%. The editing frequencies of the Pat_B05G005800 and Pat_A06G005300 genes were 25 and 12.5%, respectively, while that of the Pat_B06G005200 and Pat_U0021200 genes was only 6.25% (Fig. 1g). These results indicated that PcUbi-pEGCas9 successfully induces allele genome-editing in tetraploid patchouli. Sequencing of all mutant plants revealed four distinct genetic modifications: insertion mutation, substitution mutation, deletion mutation, and combination mutation. Deletion was the most frequent mutation type, ranging from 93.09 to 94.77% across these *PatPDS* alleles. Most of the mutations were short deletions, with 5-bp nucleotide deletion as the most frequent mutation type (29.09%) at Pat_A05G005500, 3-bp deletions at Pat_B05G005800 (32.64%), and 2-bp deletions at Pat_A06G005300 (28.23%), Pat_B06G005200 and Pat_U0021200 (44.83%) (Fig. 1h–k). Besides introducing short deletions in patchouli, the PcUbi-pEGCas9 could induce large-fragment deletions (>10 bp) with the frequencies of 18.31% (Pat_A05G005500), 11.05% (Pat_A06G005300), 18.90% (Pat_B05G005800), and 6.60% (Pat_B06G005200 and Pat_U0021200) (Fig. 1h–k). Furthermore, we further confirmed the long-fragment editing events by Sanger sequencing, and the large deletions of 16-, 46- and 92-bp were validated at the target locus of line 5, line 10, and line 11, respectively (Fig. 1l).

In conclusion, the current work provided an efficient and stable genetic transformation system and confirmed the high efficiency of this CRISPR/Cas9 system in targeted mutation for tetraploid patchouli. The method described in this research has broad application prospects in genome editing, genetic improvement, germplasm innovation, and functional genomics of patchouli.

## Acknowledgements

The research was supported by the Research and Development Program in Key Areas of Guangdong Province (No. 2021B0707010010), the Scientific Innovation Strategy-Construction of High-level Academy of Agriculture Science (R2019PY-JX003), the Director’s Fund of Crop Research Institute of Guangdong Academy of Agricultural Sciences/Open Fund of Guangdong Provincial Key Laboratory of Crops Genetics and Improvement (202206), and the Special Fund for Introducing Scientific and Technological Talents of Guangdong Academy of Agricultural Sciences (R2020YJ-YB3003).

## Author contributions

S.Q.X., J.H.W., and S.H.Y. designed and supervised the project, S.Q.X., F.Z., J.Y.L., and S.K.C. prepared the samples, S.Q.X., F.Z., and J.Y.L. performed the experiments, S.Q.X., F.P.L., and J.H.W. analyzed the data. S.Q.X. wrote the paper and S.Q.X., F.P.L., S.H.Y., and J.H.W. revised the paper. All authors read and approved the final manuscript.

## Data availability

The data sets supporting the conclusions of this article are available in the Figshare database under the accession 10.6084/m9.figshare.24850635.

## Conflict of interest

The authors declare no conflict of interest.
